# Biosorption Potential of Microbial and Residual Biomass of *Saccharomyces pastorianus* Immobilized in Calcium Alginate Matrix for Pharmaceuticals Removal from Aqueous Solutions

**DOI:** 10.3390/polym14142855

**Published:** 2022-07-13

**Authors:** Lăcrămioara Rusu, Cristina-Gabriela Grigoraș, Andrei-Ionuț Simion, Elena-Mirela Suceveanu, Bogdan Istrate, Maria Harja

**Affiliations:** 1 Faculty of Engineering, “Vasile Alecsandri” University of Bacau, 157 Calea Mărăşeşti, 600115 Bacau, Romania; asimion@ub.ro (A.-I.S.); mirela.suceveanu@ub.ro (E.-M.S.); 2 Mechanical Engineering Faculty, “Gheorghe Asachi” Technical University from Iasi, 43 Mangeron Blvd., 700050 Iasi, Romania; bogdan_istrate1@yahoo.com; 3 Faculty of Chemical Engineering an Environmental Protection “Cristofor Simionescu”, “Gheorghe Asachi” Technical University from Iasi, 71 A Mangeron Blvd., 700050 Iasi, Romania

**Keywords:** *Saccharomyces pastorianus*, ethacridine lactate, residual biomass, immobilization, biosorption, kinetic models, equilibrium isotherms

## Abstract

Two types of biosorbents, based on *Saccharomyces pastorianus* immobilized in calcium alginate, were studied for the removal of pharmaceuticals from aqueous solutions. Synthetized biocomposite materials were characterized chemically and morphologically, both before and after simulated biosorption. Ethacridine lactate (EL) was chosen as a target molecule. The process performance was interpreted as a function of initial solution pH, biosorbent dose, and initial pharmaceutical concentration. The results exhibited that the removal efficiencies were superior to 90% for both biosorbents, at the initial pH value of 4.0 and biosorbent dose of 2 g/L for all EL initial concentrations tested. Freundlich, Temkin, Hill, Redlich-Peterson, Sips, and Toth isotherms were used to describe the experimental results. The kinetic data were analyzed using kinetic models, such as pseudo-first order, pseudo-second order, Elovich, and Avrami, to determine the kinetic parameters and describe the transport mechanisms of EL from aqueous solution onto biosorbents. Among the tested equations, the best fit is ensured by the pseudo-second-order kinetics model for both biosorbents, with the correlation coefficient having values higher than 0.996. The many potential advantages and good biosorptive capacity of *Saccharomyces pastorianus* biomass immobilized in calcium alginate recommend these types of biocomposite materials for the removal of pharmaceuticals from aqueous solutions.

## 1. Introduction

In recent decades, discoveries in the field of pharmaceuticals have revolutionized both human and veterinary medicine [1,2,3]. At the same time, the prescription of drugs has changed significantly [4,5,6] and led to an increased use of pharmaceuticals. Thus, the amount of pharmacologically active compounds used to treat and prevent disease can be estimated at thousands of tons per year [1,7].

The global consumption of drugs is directly reflected in their presence in various environmental matrices, including the aquatic environment [1,4].

The pollution of surface waters, groundwater, and implicitly drinking water with pharmaceuticals and their transformation products can originate from different sources: wastewater treatment plant effluents, uncontrolled landfills leachates, pharmaceutical industry, hospitals, livestock feed, inappropriate disposal of unused drugs, or used containers, etc. [1,8,9,10]. Among them, wastewater treatment plant effluents are considered the main source, due to the fact that, in most cases, these substances are not eliminated and are detected in treated water [1,8,11,12].

The presence of pharmaceuticals in aquatic environment represents a potential risk for human health and living organisms that inhabit in this environment. Therefore, many studies have mentioned the need to develop technologies or processes that allow for the complete elimination of pharmaceutical residues from wastewater before they are discharged into the environment [1,8].

Numerous techniques have been used for the removal of pharmaceuticals from aqueous matrices, including membrane separation, ozonation, flocculation, advanced oxidation, photocatalysis, microbial degradation, electrochemical processes, and adsorption [13,14,15,16,17,18,19,20,21,22,23]. These approaches are based on physical, chemical, or biological processes and differ in effectiveness, sustainability, costs, etc.; they have various advantages and disadvantages. Of these, the adsorption process is the most promising option for removing pharmaceutical compounds from aqueous solutions. Activated carbon is the most common adsorbent, due to its effectiveness and versatility. However, it is not advantageous for sorption, due to its high cost [13,15]. In this context, the use of biological materials (biomass) as absorbents is becoming an important alternative.

Considering the previous information, the biomass can be alive or dead, and its use as a biosorbent would have more possibilities to remove a greater number of pollutants [24,25,26].

Currently, there is a growing interest in the use of microorganisms as a basic material for the development of biosorbents, due to their good sorption properties.

Thus, different species of fungi, bacteria, yeast, and microalgae have been tested for the removal of many types of pollutants with promising results [24,27,28,29].

The microbial biomass can be applied directly or immobilized/encapsulated in different matrices, such as chitosan, alginate, etc. [30,31,32,33]. Immobilization/encapsulation techniques allow for the microbial biomass to be easy separated from effluents with low cost and increase their mechanical resistance. In this context, for biosorption techniques, research is now focusing on the development of more complex systems, with the use of biocomposite materials with new characteristics.

The *Saccharomyces pastorianus* (lager yeast) strain is an interspecies hybrid, between *Saccharomyces cerevisiae* and *Saccharomyces eubayanus*, used in large quantities in brewing industry [34].

Residual biomass of *Saccharomyces pastorianus*, which is considered a second-largest by-product of the brewing industry, contains significant residual carbohydrates, proteins, aminoacids, lipids, minerals, and enzymes, and it is still investigated for obtaining high added-value products [35]. If we consider the aspects mentioned above, it turns out that this residue seems to meet all the requirements for use in obtaining a viable biosorbent: it is safe, low-cost, and available throughout the year in large volumes [31].

Ethacridine lactate (2-ethoxy-6,9-diaminoacridine monolactate monohydrate) is an acridine derivate with antiseptic action, which is indicated for the treatment of Gram-positive bacterial infections. It is widely used in the local treatment of inflammatory or ulcerative conditions of the skin. In many countries, the drug was in clinical practice for the second trimester termination of pregnancy in solutions of 0.1% [36,37].

Due to the fact that it is an effective antibacterial drug, it is use in the oral treatment of enteric disease, such as diarrhea and shigellosis. In the case of orally administered EL, is almost completely (99%) excreted in the feces [38].

EL is considered a hazardous substance according to Occupational Safety and Health Administration Organization from United States of America, with acute and chronic health effects and high toxicity for aquatic organisms [39].

Although EL is a drug used in large quantities worldwide, there are only few studies discussing the possibility of removing it from aqueous solutions. Talman et al. [40,41] used bentonite and activated carbon as adsorbents for the removal of this pharmaceutic compound.

To the best of our knowledge, the application of *Saccharomyces pastorianus* biomass immobilized/encapsulated in natural polymers matrices as a biosorbent for pharmaceuticals removal from aqueous solutions has been mentioned in our previous papers [30,31].

In the present research, two types of biosorbents were synthetized and investigated for the selected target molecule, in order to evaluate the biosorption capacity to remove pharmaceuticals from aqueous media.

The goal of this study was to compare the biosorption capacities of microbial biomass and residual microbial biomass of *Saccharomyces pastorianus* immobilized in calcium alginate for EL removal from aqueous solutions in a batch system, firstly and secondly to correlate the experimental results using a mathematical approach.

In this context, several adsorption isotherms and kinetic models were investigated to describe the experimental results. Additionally, the equilibrium and kinetic parameters of the biosorption process of ethacridine lactate were determined and discussed.

This information will contribute to the biosorption database and help to protect natural ecosystems in an economical way.

## 2. Materials and Methods

### 2.1. Reagents and Analytical Procedure

Reagents required for conducting the experiments were of analytical quality and did not undergo any treatment or purification.

Ethacridine lactate was purchased from Merck (Darmstadt, Germany). Hydrochloride acid, sodium chloride, and ethanol were delivered by Chemical Company (Iași, Romania). Sodium hydroxide and calcium chloride were bought from Chempur (Piekary Ślaskie, Poland). Sodium alginate (low viscosity grade) was procured from BUCHI Laboratortechnik AG (Flawil, Switzerland).

*Saccharomyces pastorianus*, in the form of dried and residual biomass, were a gentle donation of the brewing company Albrau (Onești, Romania).

Distilled water was used to prepare all the solutions. NaOH (0.1 M) or HCl (0.1 M) were employed in pH corrections.

A stock solution of Ethacridine lactate with a concentration of 500 mg/L was firstly prepared and kept at 4 °C in a closed vessel. Subsequent dilutions (1 mg/L to 60 mg/L) were obtained; their absorbance was recorded at a wavelength of 431 nm on a UV1280 spectrophotometer (Shimadzu, Tokyo, Japan) and served to plot the calibration curve.

All the experiments were performed in triplicate.

### 2.2. Biosorbent Preparation and Characterization

#### 2.2.1. Synthesis of Biosorbent Containing *Saccharomyces pastorianus* Dried Biomass

In a sodium alginate solution (1%) prepared with hot distilled water, dried biomass of *Saccharomyces pastorianus* was added, in order to obtain a suspension with 5% (d.w.) concentration. After complete homogenization, the mixture was dropped in a calcium chloride solution (2%). The resulted beads (called SPA 5%) were carefully washed with CaCl_2_ 2% and then kept in a fresh similar solution for 24 h at 4 °C. The storage solution was removed washing before starting the biosorption experiments.

#### 2.2.2. Synthesis of Biosorbent Containing Residual Biomass of *Saccharomyces pastorianus*

Residual biomass of *Saccharomyces pastorianus* was thawed, repeatedly washed, decanted, and centrifuged (2500 rpm, 2 × 10 min) in a Quirumed 80-2A laboratory centrifuge (Jintan City, China). A specific amount of the residual biomass, thus prepared, was introduced in a solution obtained by dissolving sodium alginate (1%) in phosphate buffer (pH 7), with the scope reaching a final concentration of 5%. A thorough homogenization was ensured. As in the case of SPA 5%, the mixture was suspended in a calcium chloride solution (2%). The resulted beads (called SPRBA 5%) were also washed with CaCl_2_ 2% and then kept in a fresh similar solution for 24 h at refrigerator (4 °C). The storage solution was removed washing before starting the biosorption experiments.

#### 2.2.3. Biosorbents Characterization (SEM, FTIR, Point of Zero Charge)

A SEM Quanta 200 3D (FEI Europe B.V., Eindhoven, The Netherlands) apparatus, equipped with an energy-dispersive X-ray system, was employed for the scanning electron microscopy analysis (SEM). To this end, the biosorbents were firstly dried at 50 °C for 2 h in an Air Performance AP60 hot-air oven, (Froilabo, Paris, France) and then positioned to stubs with double adhesive carbon discs. Normal secondary electron mode (SE), in low vacuum, was used. A large field detector (LFD) with accelerating voltage of 20 kV, working distance of 14.6–15.5 mm, and spot size of 5 ensured the detection. The magnification range was 1 mm to 10 μm.

FTIR spectra were registered between 4000 and 400 cm^−1^ (32 sample/background scans; 4 cm^−1^ resolution) with a Nicolet iS50 FTIR spectrometer (Thermo Scientific, Dreiech, Germany) coupled with an ATR accessory. The ATR cleaning was made with ethanol after each spectrum. The reference background spectrum was recorded with air.

For the determination of the point of zero charge (pH_PZC_) value, 0.4 g of each biosorbent were mixed for 24 h on magnetic plates at room temperature with 20 mL of 0.1 M NaCl solutions, with initial pH adjusted between 2 and 12. A portable pH meter (Dostmann KLH9.1, 0–14 pH, Carl Roth, Karlsruhe, Germany) was used for measurements at the beginning (pH_i_) and end of the experiments (pH_f_). A plot with the recovered data was then drawn.

### 2.3. Biosorption Process (pH, Biosorbent Dose, Initial Contaminant Concentration)

The experimental setup was initiated by studying the effect of the initial pH of EL solutions (60 mg/L). Its value was varied between 2 and 10 with 1 g/L of biosorbents beads. The tested biosorbents doses were from 1 to 3 g/L. Finally, EL initial concentration changes from 20 to 60 mg/L were considered. All the experiments were conducted in triplicate for 24 h at ambient temperature.

The remaining EL concentrations were established by reading the samples absorbance at 431 nm against the calibration curve.

Removal efficiency (*R*, %) and biosorption capacity (*Q_e_*, mg/g) calculus were realized with the Equations (1) and (2):

(1) $$R=\frac{C_{0}-C_{e}}{C_{0}}\cdot 100$$

(2) $$Q_{e}=\frac{(C_{0}-C_{e})\cdot V}{m}$$

where *C*_0_ and *C_e_* are EL initial and at equilibrium concentrations (mg/L), *m* is the biosorbent dose (g/L), and *V* is the EL volume (L).

### 2.4. Kinetics and Equilibrium Isotherms

Various extensively used nonlinear kinetic models (pseudo-first-order, pseudo-second-order, Elovich, and Avrami) and equilibrium isotherms (Freundlich, Temkin, Hill, Redlich-Peterson, Sips, and Toth) existing in CAVS adsorption evaluation software (Federal University of Paraná, Curitiba, Paraná, Brazil) were applied for the validation of the biosorption conduct of ethacridine lactate by the two biosorbents obtained by immobilization of *Saccharomyces pastorianus* dried biomass and residual biomass on inert matrix of calcium alginate.

Nonlinear equations and constants significance for each kinetic and equilibrium isotherm model are presented in Table 1 and Table 2, respectively.

### 2.5. Statiscal Analysis

Experimental data were analyzed via CAVS adsorption evaluation software. Root mean square error (*RMSE*), Marquardt’s percent standard deviation (*MPSD*), hybrid fractional error function (*HYBRID*), chi-square (*χ*^2^), and coefficient of determination (*R*^2^) were used for the evaluation of the goodness of fit. Table 3 includes the specific equations of these statistical parameters.

## 3. Results and Discussion

### 3.1. Biosorbents Preparation and Characterization

Natural and synthetic polymers are known as non-toxic, biodegradable, and highly available matrices. Composed of β-d-mannuronic acid (1-4)-linked and α-l-guluronic acid, sodium alginate is one such polymer. It shows the important ability of forming a network structure with divalent cations (e.g., calcium) [42]. In our case, its use ensured a good immobilization of *Saccharomyces pastorianus* dried biomass or residual biomass, thus allowing us to prepare new biosorbents.

**Figure 1:**
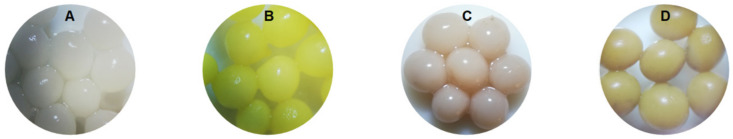
Photographs of synthesized biosorbents ((**A**,**B**)—SPA 5%; (**C**,**D**)—SPRBA 5%) before (**A**,**C**) and after (**B**,**D**) biosorption of ethacridine lactate.

The aspect of the obtained beads (SPA 5%, SPRBA 5%) is revealed in Figure 1. As can be seen, they possess both a whitish hue, lighter for SPA 5% and darker for SPRBA 5%. This difference can be attributed to the fact that the residual biomass of *Saccharomyces pastorianus* contains different impurities, which resulted from the brewing process. A regular, spherical form is to be observed, the mean diameters being similar with values of 3.339 ± 0.020 mm for SPA 5% and 3.226 ± 0.029 mm for SPRBA 5%.

**Figure 2:**
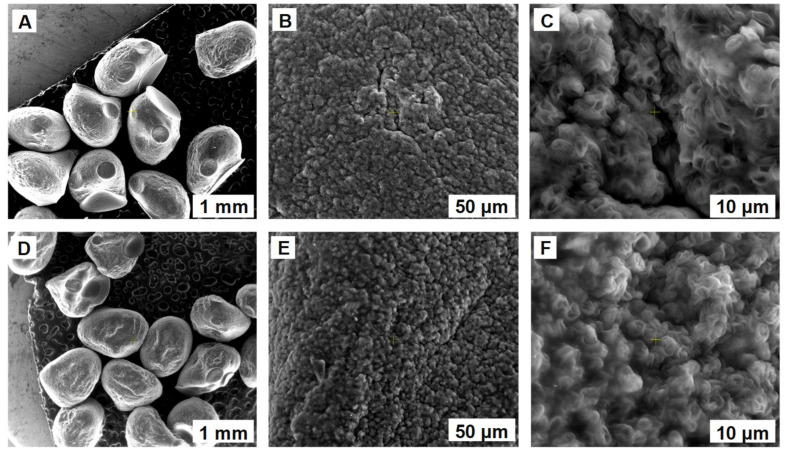
SEM images of SPA 5% biosorbent prepared before (**A**–**C**) and after (**D**–**F**) biosorption of ethacridine lactate from aqueous solution.

**Figure 3:**
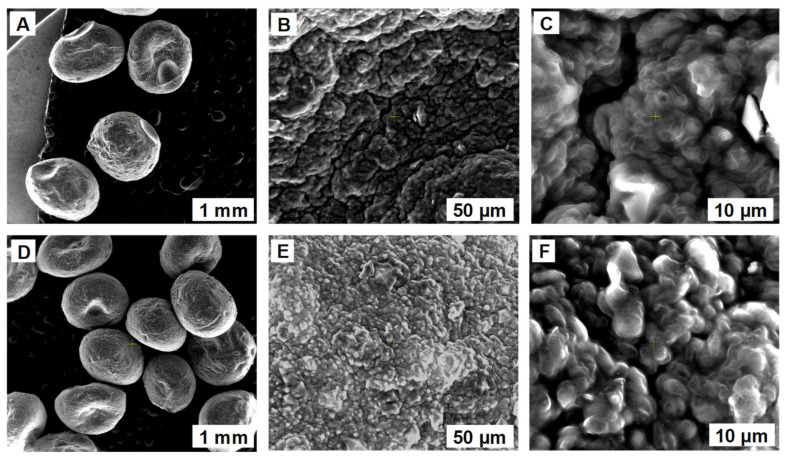
SEM images of SPRBA 5% biosorbent prepared before (**A**–**C**) and after (**D**–**F**) biosorption of ethacridine lactate from aqueous solution.

Scanning electron microscopy served to analyze the morphological characteristics of the synthesized biosorbents. Figure 2 and Figure 3 display images with the beads before and after the biosorption of ethacridine lactate from aqueous solutions. The external surface seems to be a smooth one, presenting only a few irregularities, caused by the dripping process. The internal morphology is similar for both SPA 5% and SPRBA 5% biosorbents. A uniform, porous structure can be noticed, with higher tendencies of agglomeration after biosorption. The recorded modifications confirm the retention of the tested pollutant.

**Figure 4:**
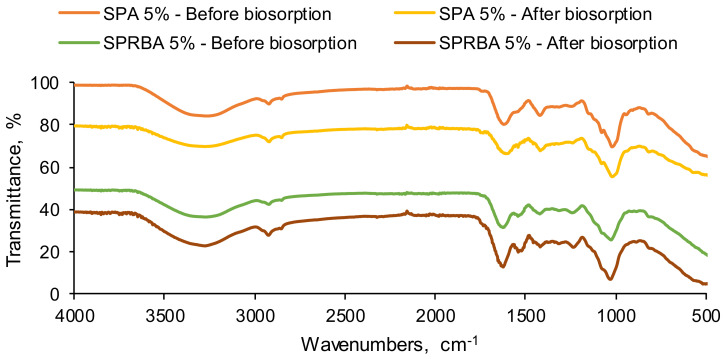
FTIR spectra of SPA 5% and SPRBA 5% biosorbents before and after ethacridine lactate biosorption.

The investigation of functional groups existing in SPA 5% and SPRBA 5% biosorbents, before and after the EL biosorption, was piloted by FTIR analysis. Spectra exposed in Figure 4 reveal the presence of the inert matrix of alginate. At high frequencies (3000 to 3200 cm^−1^), vibrations of hydroxyl were visible. At 2920 cm^−1^, the aliphatic stretching vibration of –CH was detected [43]. The bands from 1600 to 1400 cm^−1^, corresponding to the asymmetric and symmetric stretching vibrations of carboxyl ions [44] of C–O (between 1100 cm^−1^ and 900 cm^−1^) and mannuronate and guluronate residues (1030 cm^−1^) [45], are specific for the natural polymer in which the immobilization of *Saccharomyces pastorianus* dried biomass and residual biomass was realized. A –CH_2_ bending vibration close to 1000 cm^−1^ was also detectable. Similar outcomes were presented by Larosa et al. [46], who studied bare and tannase-loaded calcium alginate beads and reported comparable assignments for the collected spectra. Peaks of 1630 and 1540 cm^−1^ can be accredited to amide I and amide II. From approximatively 1300 and 1200 cm^−1^, bands for amide III (proteins) and PO_2_^−^ (phosphorylated proteins and phospholipids), possibly caused by the yeast incorporated into the polymeric material, appeared [47]. These facts sustain the idea that dried biomass and residual biomass were, respectively, well-incorporated in the resulted adsorbent beads.

When examining the spectra collected after adsorption, it can be appreciated that the signals of the target pollutant were overlapped by functional groups of the biosorbents. It is the case of for the bands encountered between 3500 and 3100 cm^−1^, specific for the N–H asymmetric and symmetric stretching vibrations of aromatic amine and hydrogen-bonded N–H bands. Along with this, the peak recorded at approximatively 1630 cm^−1^ is also characteristic for the C=N vibrations that exist in the acridine ring of the ethacridine lactate [48]. As consequence, it can be concluded that the prepared biosorbents were able to adsorb the contaminant from its aqueous solutions.

The characterization of SPA 5% and SPRBA 5% biosorbents was completed with the determination of the point of zero charge. This point designates the pH of a solution at which there is an equality between the charge of the positive and negative surface sites; therefore, the biosorbent surface charge is null. pH_PZC_ serves to establish whether the surface charge is negative (pH > pH_PZC_) or positive (pH < pH_PZC_) [49].

**Figure 5:**
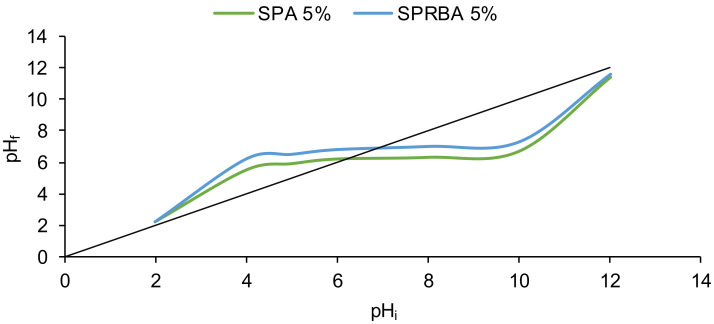
pH_PZC_ of SPA 5% and SPRBA 5% biosorbents (pH_f_—final pH; pH_i_—initial pH).

As shown in Figure 5, similar shapes were obtained for both synthesized biosorbents. At the beginning of the curves, an increase of pH_f_ from 2.20 to 5.50 for SPA 5% and 2.20 to 6.20 for SPRBA 5% was observed with the increase of pH_i_ from to 2 to 4.

A pH of the initial solution set at 12 led to a new increase of pH_f_ at a maximum of 11.40 for SPA 5% and 11.60 for SPRBA 5%. Between these two periods, a plateau at about 5.90–6.70 for SPA 5% and about 6.20–6.70 for SPRBA 5% was reached when the pH_i_ varied from 4 to 10. The plateau represents the range of initial pH of EL solutions in which buffer properties can be registered. The addition of an acid or base in the plateau will not influence the pH_f_. Therefore, it can be considered that its value will remain close to that of pH_PZC_. The biosorbents surfaces are positively charged when pH_i_ is below 4 and negatively charged when pH_i_ is higher than 10.

For the biosorbents obtained, pH_PZC_ was established at 6.20 for SPA 5% and 6.80 for SPRBA 5%.

### 3.2. Impact of pH, Biosorbent Dose and EL Initial Concentration on the Biosorption Process

The first parameter chosen for the study of the effect of biosorption conditions was the pH of EL solution. Volumes of 30 mL of EL solutions with concentration of 60 mg/L and pH adjusted from 2 to 10 were put in contact with biosorbents, with their concentration being of 1 g/L.

**Figure 6:**
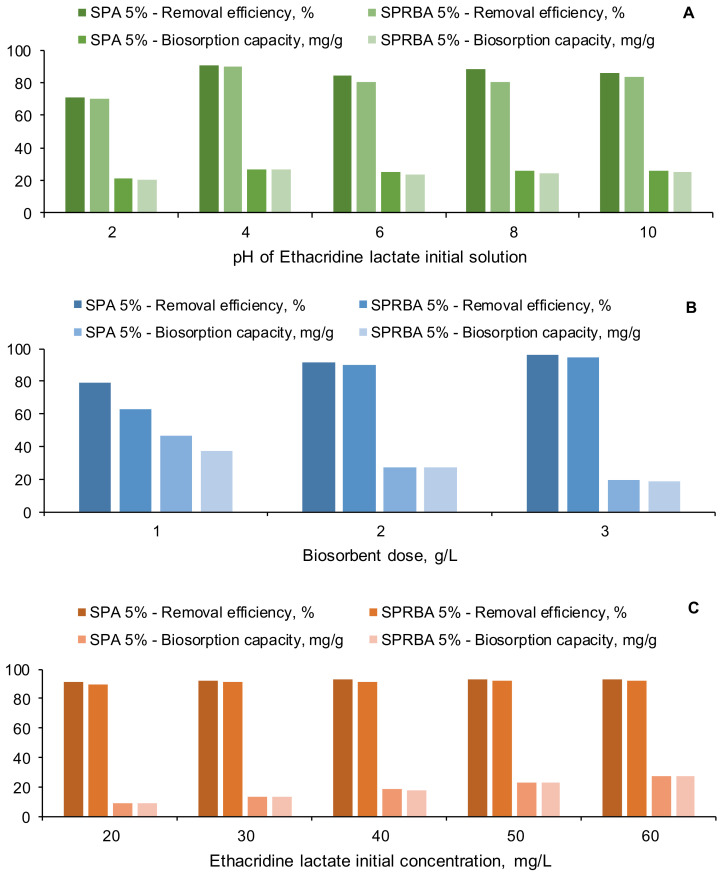
Influence of parameters on the biosorption process (**A**): effect of EL solution initial pH (volume of EL solution: 30 mL; concentration of EL solution: 60 mg/L; biosorbent dose: 1 g/L); (**B**): effect of biosorbent dose (volume of EL solution: 30 mL; concentration of EL solution: 60 mg/L; pH of EL solution: 4); (**C**): effect of EL solution initial concentration (volume of EL solution: 30 mL; pH of EL solution: 4; biosorbent dose: 2 g/L) (SPA 5%—*Saccharomyces pastorianus* immobilized on calcium alginate 5%; SPRBA 5%—*Saccharomyces pastorianus* residual biomass immobilized on calcium alginate 5%).

According to data illustrated in Figure 6A, comparable values were collected independently of the biosorbent tested. The same trend could be detected with the lower results at pH 2 and higher, but similar, ones for pH, ranging from 4 to 10. These results are consistent with the facts related to the pH_PZC_, according to which, between pH 4 and pH 10, the EL solution possesses buffer properties. In one of our previous studies [31], we explained that EL has the ability to dissociate in water being retained by the biosorbents which, at acid pH, have surfaces charged positively. Our supposition is similar to those of Okada et al. [50] and Talman et al. [40], who also concluded that EL dissociates in aqueous solutions and the pH of EL has not a great impact on the adsorption process. The highest removal efficiencies and biosorption capacities were obtained at pH 4, and they were of 91.05% and 26.72 mg/g for SPA 5% and 89.93% and 26.76 mg/g for SPRBA 5%. Thus, the pH 4 was considered appropriate for further biosorption process development.

In a second step of our experimental study, we kept constant the pH of EL solution and its concentration, and we changed the amount of the biosorbent added from 1 g/L to 3 g/L. Figure 6B depicts the evolution of the removal efficiencies and of biosorption capacities in the established settings. The most convenient results (R = 91.73%, q = 27.47 mg/g for SPA 5% and R = 90.27%, q = 26.97 mg/g for SPRBA 5%) were acquired when a concentration of 2 g/L of biosorbent was used. When the biosorbents were added in a concentration of 3 g/L, the removal efficiencies were higher, with approximatively 5%. The difference was not considered significant enough to justify the use of a double dose of biosorbent.

The last studied parameter with influence on the biosorption process was the EL solution initial concentration. It varied from 20 to 60 mg/L. As can be seen in Figure 6C, for all the tested EL concentrations, the removal efficiencies were superior to 90% for both biosorbents. This allows us to conclude that, regardless the type of *Saccharomyces pastorianus* biomass (dried or residual) used for biosorbents production, the results in retaining the pollutant from aqueous solutions are very promising.

### 3.3. Biosorption Kinetics

The kinetics experiments were carried out at pH 4, with EL volumes of 30 mL and biosorbents doses of 2 g/L. The initial concentrations of the target contaminant varied from 20 to 60 mg/L. The samples were recovered and analyzed. Among the numerous existing kinetic models, nonlinear forms of pseudo-first-order, pseudo-second-order, Elovich, and Avrami were tested, in order to find out the most suitable ones for describing the EL biosorption.

Pseudo-first-order kinetics refers to the rate of adsorption in a liquid system. It permits to establish the values of the time-scaling factor, which indicates the speed with which the system attains the equilibrium state and of the quantity of the compound adsorbed at equilibrium [51]. Pseudo-second-order kinetics is based on the hypothesis that the adsorption involves different mechanisms, such as chemical and electrostatic interactions occurring between the adsorbent molecule and the biosorbent surface [52]. It also stipulates that the concentration of the target compound directly influences the number of the occupied biosorbent sites [53]. Elovich kinetics model sustains that the activation energy will increase when the biosorption time raises and adsorbent material is characterized by a heterogeneous surface [54]. Avrami model theory presumes that the reaction between the adsorbate and adsorbent takes place on the surface of the active sites. The main parameters affecting this model are represented by the constants *k_Av_* and *n_Av_*, with the latter indicating alterations of the adsorption mechanisms in time and with temperature [55,56].

**Figure 7:**
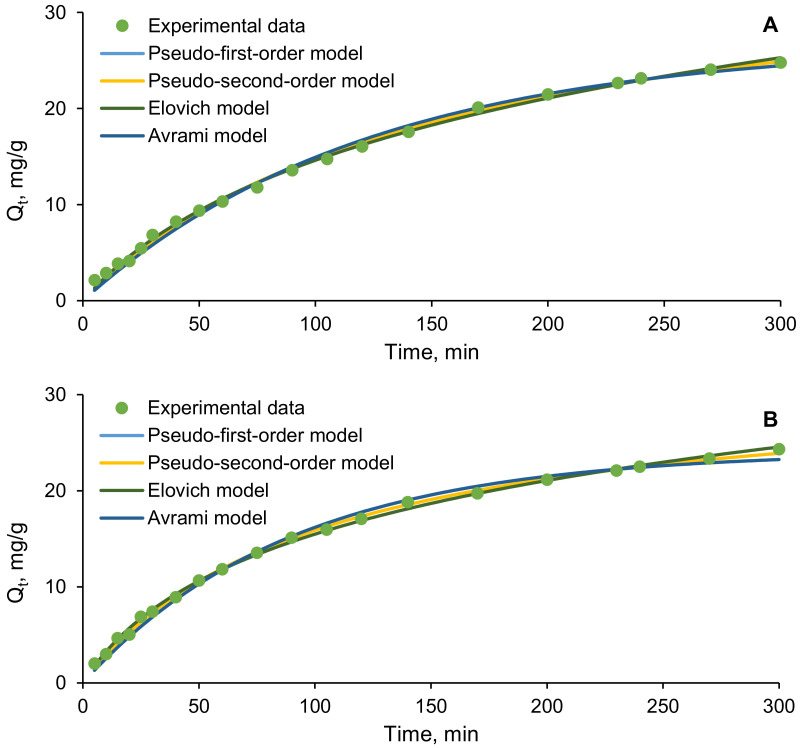
Kinetic models for the biosorption of EL on synthesized biosorbents ((**A**)—SPA 5%; (**B**)—SPRBA 5%) (*Q_t_*—concentration on the solid phase at time *t*).

Figure 7 exemplifies the above-mentioned kinetics models fitted to the experimental data recorded, in the case of biosorption conducted with 30 mL of EL solution having pH 4, a pollutant initial concentration of 60 mg/L, and a biosorbent dose of 2 g/L. The contact time was set at 300 min.

The kinetic parameters of the biosorption process recovered from the model plots are given in Table 4 (for SPA 5%) and in Table 5 (for SPRBA 5%).

Statistical error functions for the kinetics are reported in Table 6 (for SPA 5%) and in Table 7 (for SPRBA 5%).

Among the tested equations, the pseudo-first-order and pseudo-second-order kinetics are the closest to the experimental data. Nevertheless, the best fit is ensured by the second model. For both the obtained biosorbents, the correlation coefficient has values higher than 0.996 and reduced values for all the statistical error functions applied. These findings imply that there was a chemical adsorption that affected the adsorbent rates and the uptake of ethacridine lactate on the composite materials prepared by immobilization of dried biomass or residual biomass on the natural polymeric matrix of calcium alginate. The same observation was made by Adeola et al. [57] who studied the adsorption of efavirenz and nevirapine on graphene wool and arrived to the conclusion that the pseudo-second-order kinetic model is the most suitable for describing the interactions between the adsorbent and the tested molecules. A chemical-controlling mechanism was established also by Altalhi et al. [58] who reported that the adsorption of doxorubicin hydrochloride on a green adsorbent follows the pseudo-second-order kinetics. *k*_2_ constant depends on the experimental conditions especially on the initial concentration in pollutant of the aqueous solution. As shown in Table 4 and Table 5, it diminishes from 0.0004 g/(mg·min) to 0.0001 g/(mg·min) with the augmentation of the concentration from 20 mg/L to 60 mg/L when the adsorption is realized on SPA 5% and from 0.0008 g/(mg·min) to 0.0003 g/(mg·min) for the same concentration range when the adsorption is realized on SPRBA 5%. The lower value of *k*_2_ indicates that the time required for the equilibrium reach is rather long. The other main parameter of the pseudo-second-order kinetic model is the quantity of the contaminant retained at equilibrium (*Q_e_*). Again, in all of our studied cases, there were no significant differences between the experimental data and those recuperated from the mathematical model.

Elovich kinetic model is recognized as being restricted to the initial period of a biosorption process, when the equilibrium is far of being reached; however, there is research explaining that this model can be interpreted as very similar with that expressed by the pseudo-second-order kinetic relation [59]. In our case, the correlation coefficients were lower for Elovich model, even though superior of 0.9920 for all the concentrations and both biosorbents. At the same time, higher values for the statistical error functions were recorded.

In all the tested situations, the constant *n_Av_* of the Avrami model has low values (<1), suggesting that the biosorption is homogeneous and does not happen with a constant evolution rate. Analogous findings were highlighted by Benedini et al. [60], who claimed that the adsorption of some antibiotic and anti-inflammatory drugs on a hydroxyapatite composite correlates with high degree of accuracy with Avrami isotherm.

### 3.4. Equilibrium Isotherms

Different equilibrium isotherms were applied on the experimental obtained data, with the purpose of finding the mechanisms of EL biosorption on the prepared biosorbents.

**Figure 8:**
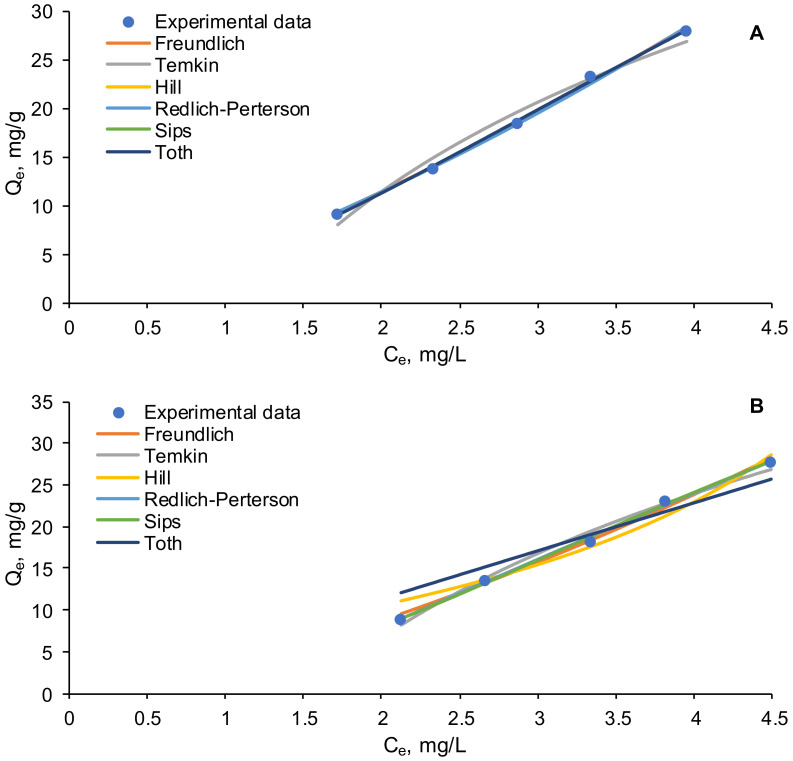
Equilibrium isotherms for the biosorption of EL on synthesized biosorbents ((**A**)—SPA 5%; (**B**)—SPRBA 5%).

Figure 8 presents a graphical representation of the tested isotherms. According to the statistical analysis, most of them are fairly pertinent to describe the experimental data. They follow the sequences Sips = Hill > Freundlich > Riedlich-Peterson > Temkin > Toth for the biosorption of EL on SPA 5% biosorbent and Riedlich-Peterson > Sips > Frendlich > Temkin > Hill > Toth, when the biosorption is conducted with SPRBA 5% biosorbent.

Freundlich isotherm refers to the multilayer adsorption on adsorbents with heterogeneous surface [61]. The *K_F_* Freundlich constant is higher than 1 and indicates that physical adsorption of ethacridine lactate occurred on both adsorbents. The negative *1/n* ratio discloses a favorable adsorption.

Temkin model ignores the high and low concentrations of the target pollutant compound in the liquid phase and presumes that the adsorption is a multilayer process. The reduced values of *b* Temkin constant reveals that the interactions between the biosorbents and the target molecule are weak, also sustaining the physisorption [62].

Hill equation considers that the adsorbate has the ability to bind on one site of the biosorbent influencing the other sites. The positive *n_H_* constant shows the cooperativity phenomenon when SPA 5% biosorbent was used, while the negative value registered for SPRBA 5% indicates a negative cooperativity.

Redlich-Peterson and Sips isotherms are a mix of Langmuir and Freundlich models and are used in homogeneous and heterogeneous adsorption.

Toth model extends the utility of Langmuir isotherm to heterogeneous systems and is based on the fact that the adsorption energy of the adsorption sites is lower than the mean energy [63].

Talman et al. [40] used the Langmuir and Freundlich models to analyze the equilibrium, in the case of EL removal using activated carbon and bentonite as adsorbents. Comparable results to those of this study were obtained for the use of activated carbon (q_predicted_ = 68.67 mg/g), while the bentonite possess much higher ability to retain EL (q_predicted_ = 721.07 mg/g). Obradovic et al. [64] used functionalized minerals to retain ibuprofen and diclofenac sodium and concluded that the Freundlich isotherm well-fit the experimental data. On the contrary, in other paper [65], the authors declared that the Freundlich model was not able to justify the results recorded for the adsorption of acetaminophen on an hybrid adsorbent composed of polyaniline and chitosan. Another study conducted for the removal of various dyes, drugs, and metal from aqueous solutions by adsorption on a polymeric composite established that Langmuir and Temkin models closely follow the records [66]. Even though, Langmuir isotherm is of the most known model used for describing the adsorption behavior, in our case, it does not fit the experimental results (data not shown), which let us conclude that the biosorption process is not a homogenous monolayer one.

The parameters of these isotherms are given in Table 8 and Table 9, and their statistical error functions are presented in Table 10 (for SPA 5%) and in Table 11 (for SPRBA 5%).

## 4. Conclusions

The present work was focused on studying the potential biosorption capacity of biosorbents, based on *Saccharomyces pastorianus* biomass immobilized in calcium alginate to remove EL from aqueous solutions. A comparative study of biosorption between two types of biosorbents obtained from the pure microorganism and residual biomass of the same yeast species was performed.

Synthesized biosorbents were characterized, in terms of morphology (SEM), functional groups (FTIR), particle size, and point of zero charge. The obtained beads, named SPA 5% and SPRBA 5%, showed regular, spherical forms; they had similar mean diameters with values of 3.339 ± 0.020 mm for SPA 5% and 3.226 ± 0.029 mm for SPRBA 5%.

Analyzing the FTIR spectra before and after biosorption, it can be appreciated that the signals of the target pollutant were overlapped by functional groups of the biosorbents. The recorded SEM images confirmed the retention of the tested pollutant. The values for pH_PZC_ were established at 6.20 for SPA 5% and 6.80 for SPRBA 5%.

Different experiments were effectuated for establishing the influence of main biosorption process parameters, initial pH, biosorbent dose, and EL initial concentration. It was found that, in both cases (SPA 5% and SPRBA 5%), the biosorption processes presented pH and biosorbent dose dependence. It was obtained a removal efficiency over 90% and biosorption capacity around 27 mg/g for both biosorbents at the initial pH value of 4.0 and biosorbent dose of 2 g/L for all EL initial concentrations tested.

The kinetics approach was performed by testing nonlinear forms of pseudo-first-order, pseudo-second-order, Elovich, and Avrami, in order to find out the most suitable ones for describing the EL biosorption. The experimental data of EL removal were best fitted by pseudo-second-order model (*R*^2^ > 0.996) for both the obtained biosorbents.

Taking the fact that the equilibrium isotherms are considered a powerful tool in the practical design and operations of adsorption processes into account, in this study, in order to analyze and fit the experimental data, the Freundlich, Temkin, Hill, Redlich–Peterson, Sips, and Toth models were used. According to the obtained correlation coefficients values, most of them are fairly pertinent to describe the experimental data. They follow the sequences Sips = Hill > Freundlich > Redlich-Peterson > Temkin > Toth for the biosorption of EL on SPA 5% biosorbent and Redlich-Peterson > Sips > Freundlich > Temkin > Hill > Toth when the biosorption is conducted with SPRBA 5% biosorbent.

As a result, the biocomposite materials synthesized in this research work by immobilization of *Saccharomyces pastorianus* biomass on calcium alginate, which can be considered low-cost, easy to use, and eco-friendly biosorbents. They possess good biosorptive ability and can be recommended for removal of pharmaceuticals from aqueous solutions.

In our opinion, this study opens the way to a new valorization direction of the residual microbial biomass resulting from various fermentation processes.

Future research will aim for the simultaneous removal of several types of persistent organic pollutants, with the ultimate goal being the integration of this type of process in the technological flow of wastewater treatment.

## Figures and Tables

**Table 1:** Nonlinear equations of kinetic models.

Kinetic Model	Equation	Parameters Significance ^1^
Pseudo-first-order	$Q_{t}=Q_{e}\cdot \left(1-e^{-k_{1}\cdot t}\right)$	*k*_1_ is the pseudo-first-order constant rate, 1/min *t* is the contact time, min
Pseudo-second-order	$Q_{t}=\frac{k_{2}\cdot Q_{e}^{2}\cdot t}{\left(1+k_{2}\cdot Q_{e}\cdot t\right)}$	*k*_2_ is the pseudo-second-order constant rate, g/(mg·min) *t* is the contact time, min
Elovich	$Q_{t}=\frac{1}{\beta }\cdot \ln\left(\alpha \cdot \beta \cdot t\right)$	*β* is the extent of surface coverage and activation energy for chemisorption, g/mg *α* is the initial adsorption rate, mg/(g·min) *t* is the contact time, min
Avrami	$Q_{t}=Q_{e}\cdot \left(1-e^{\left(-k_{Av}\cdot t^{n_{Av}}\right)}\right)$	*k_Av_* is the overall rate constant, 1/min *n_Av_* is parameter related to the adsorption, dimensionless *t* is the contact time, min

^1^ In all the equations, *Q_t_* is the concentration on the solid phase at time *t*, mg/g and *Q_e_* is the adsorbent capacity at equilibrium, mg/g.

**Table 2:** Nonlinear equations of equilibrium isotherms.

Equilibrium Isotherm	Equation	Constants Significance ^1^
Freundlich	$Q_{e}=K_{F}\cdot C_{e}^{1/n}$	*K_F_* is Freundlich constant, (mg/g)(L/mg)^1/*n*^ *n* is Freundlich constant, dimensionless
Temkin	$Q_{e}=\frac{R\cdot T}{b}\cdot \ln\left(K_{T}\cdot C_{e}\right)$	*R* is gas constant, R = 8.314 J/(mol K) *T* is temperature, K *K_T_* is Temkin constant, L/mg *b* is Temkin constant, J/mg
Hill	$Q_{e}=\frac{Q_{H}\cdot C_{e}^{n_{H}}}{K_{D}+C_{e}^{n_{H}}}$	*Q_H_* is Hill maximum uptake, mg/g *K_D_* is Hill constant, L/mg *n_H_* is the cooperativity coefficient of the binding interaction, dimensionless
Redlich–Peterson	$Q_{e}=\frac{Q_{R}\cdot C_{e}}{1+a_{R}\cdot C_{e}^{b_{R}}}$	*K_R_* is Redlich–Peterson constant, L/g *a_R_* is Redlich–Peterson constant, L/mg *b_R_* is Redlich–Peterson exponent, dimensionless
Sips	$Q_{e}=\frac{Q_{S}\cdot {\left(K_{S}\cdot C_{e}\right)}^{B_{S}}}{1+{\left(K_{S}\cdot C_{e}\right)}^{B_{S}}}$	*Q_S_* is Sips maximum uptake, mg/g *K_S_* is Sips constant, L/mg *B_S_* is Sips exponent, dimensionless
Toth	$Q_{e}=\frac{Q_{T}\cdot C_{e}}{{\left(a_{T}+C_{e}\right)}^{1/n_{T}}}$	*Q_T_* is Toth maximum uptake, mg/g *a_T_* is Toth constant, L/mg *n_T_* is Toth constant, dimensionless

^1^ In all the equations, *Q_e_* is the adsorbate concentration on the solid phase at equilibrium, mg/g and *C_e_* is the adsorbate concentration on the fluid phase at equilibrium, mg/L.

**Table 3:** Equations used for statistical error analysis.

Statistical Parameter	Mathematical Expression
*RMSE*	$RMSE=\sqrt{\frac{1}{n-2}\cdot \overset{n}{\underset{i=1}{\sum }}{\left(Q_{e,\;\;exp}-Q_{e,\;calc}\right)}^{2}}$
*MPSD*	$MPSD=100\cdot \sqrt{\frac{1}{n-P}\cdot {\left(\frac{Q_{e,\;exp}-Q_{e,\;calc}}{Q_{e,\;exp}}\right)}^{2}}$
*HYBRID*	$HYBRID=\frac{100}{n-P}\cdot \overset{n}{\underset{i=1}{\sum }}{\left[\frac{{\left(Q_{e,\;\exp}-Q_{e,\;calc}\right)}_{i}^{2}}{Q_{e,\;exp}}\right]}_{i}$
*χ* ^2^	$\chi^{2}=\overset{n}{\underset{i=1}{\sum }}\frac{{\left(Q_{e,\;exp}-Q_{e,\;calc}\right)}_{i}^{2}}{Q_{e,\;calc}}$
*R* ^2^	$R^{2}=\frac{\sum (Q_{e,\;calc}-{\bar{Q_{e,\;exp})}}^{2}}{\sum (Q_{e,\;calc}-{\bar{Q_{e,\;exp})}}^{2}+\sum {\left(Q_{e,\;calc}-Q_{e,\;exp}\right)}^{2}}$

**Table 4:** Kinetic parameters of the biosorption process conducted on SPA 5% biosorbent.

Kinetic Model	EL Initial Concentration, mg/L	Kinetic Parameters						
		*Q_e_*	*k*_1_	*k*_2_	*α*	*β*	*k_Av_*	*n_Av_*
Pseudo-first-order	20	8.7441	0.0072	-	-	-	-	-
	30	12.9952	0.0080	-	-	-	-	-
	40	17.8438	0.0082	-	-	-	-	-
	50	22.7148	0.0079	-	-	-	-	-
	60	26.7650	0.0081	-	-	-	-	-
Pseudo-second-order	20	12.5990	-	0.0004	-	-	-	-
	30	18.4352	-	0.0003	-	-	-	-
	40	25.2392	-	0.0002	-	-	-	-
	50	32.2967	-	0.0001	-	-	-	-
	60	37.6285	-	0.0001	-	-	-	-
Elovich	20	-	-	-	0.0781	0.2349	-	-
	30	-	-	-	0.1321	0.1636	-	-
	40	-	-	-	0.1854	0.1201	-	-
	50	-	-	-	0.2773	0.0932	-	-
	60	-	-	-	0.2822	0.0818	-	-
Avrami	20	8.7441	-	-	-	-	0.0998	0.0724
	30	12.9952	-	-	-	-	0.0788	0.1026
	40	17.8438	-	-	-	-	0.0813	0.1010
	50	22.7148	-	-	-	-	0.0911	0.0876
	60	26.6750	-	-	-	-	0.0818	0.0996

**Table 5:** Kinetic parameters of the biosorption process conducted on SPRBA 5% biosorbent.

Kinetic Model	EL Initial Concentration, mg/L	Kinetic Parameters						
		*Q_e_*	*k*_1_	*k*_2_	*α*	*β*	*k_Av_*	*n_Av_*
Pseudo-first-order	20	7.8596	0.0108	-	-	-	-	-
	30	12.2111	0.1034	-	-	-	-	-
	40	16.1200	0.0108	-	-	-	-	-
	50	20.4265	0.0107	-	-	-	-	-
	60	20.0882	0.0111	-	-	-	-	-
Pseudo-second-order	20	10.5802	-	0.0008	-	-	-	-
	30	16.5292	-	0.0005	-	-	-	-
	40	21.5583	-	0.0004	-	-	-	-
	50	27.2783	-	0.0003	-	-	-	-
	60	31.9313	-	0.0003	-	-	-	-
Elovich	20	-	-	-	0.1171	0.3088	-	-
	30	-	-	-	0.1728	0.1961	-	-
	40	-	-	-	0.2474	0.1538	-	-
	50	-	-	-	0.3147	0.1221	-	-
	60	-	-	-	0.3919	0.1059	-	-
Avrami	20	7.8596	-	-	-	-	0.0892	0.1212
	30	12.2111	-	-	-	-	0.0721	0.1434
	40	16.1200	-	-	-	-	0.1027	0.1055
	50	20.4265	-	-	-	-	0.1238	0.0871
	60	24.0882	-	-	-	-	0.1307	0.0851

**Table 6:** Statistical error functions of estimated kinetic nonlinear models for the biosorption process conducted on SPA 5% biosorbent.

Kinetic Model	EL Initial Concentration, mg/L	Statistical Error Function				
		*RMSE*	*MPSD*	*HYBRID*	*Χ* ^2^	*R* ^2^
Pseudo-first-order	20	0.1841	17.2244	1.8257	0.3317	0.9942
	30	0.1932	6.7392	1.0871	0.2092	0.9973
	40	0.2364	5.1621	1.0964	0.2139	0.9979
	50	0.3155	6.0400	1.7042	0.3290	0.9976
	60	0.5595	15.2104	7.3399	2.0171	0.9944
Pseudo-second-order	20	0.1510	19.7638	1.5343	0.2355	0.9961
	30	0.1549	9.2838	1.1011	0.1836	0.9983
	40	0.1871	4.8811	0.8149	0.1437	0.9986
	50	0.2474	5.8828	1.2903	0.2207	0.9985
	60	0.4223	12.5293	4.5434	1.2118	0.9968
Elovich	20	0.1364	23.9650	1.7086	0.2186	0.9968
	30	0.1894	13.6630	1.9533	0.2889	0.9974
	40	0.2487	7.9983	1.5949	0.2557	0.9976
	50	0.3035	8.6210	2.1413	0.3315	0.9978
	60	0.3633	10.1217	2.9546	1.2118	0.9976
Avrami	20	0.1841	17.7237	1.9332	0.3317	0.9942
	30	0.1932	6.9345	1.1511	0.2093	0.9973
	40	0.2364	5.3118	1.1610	0.2139	0.9979
	50	0.3155	6.2151	1.8044	0.3290	0.9976
	60	0.5595	15.6514	7.7718	2.0171	0.9944

**Table 7:** Statistical error functions of estimated kinetic nonlinear models for the biosorption process conducted on SPRBA 5% biosorbent.

Kinetic Model	EL Initial Concentration, mg/L	Statistical Error Function				
		*RMSE*	*MPSD*	*HYBRID*	*Χ* ^2^	*R* ^2^
Pseudo-first-order	20	0.1294	29.7826	2.3720	0.2648	0.9971
	30	0.2273	27.5194	3.2055	0.3819	0.9962
	40	0.3170	5.8542	1.5394	0.2926	0.9957
	50	0.4459	6.5103	2.5212	0.5034	0.9946
	60	0.5538	11.1238	4.7950	1.0813	0.9939
Pseudo-second-order	20	0.1393	37.4927	3.6818	0.3536	0.9966
	30	0.1722	34.5163	4.3600	0.4102	0.9978
	40	0.1734	8.0616	1.0131	0.1594	0.9987
	50	0.2201	4.6546	0.7909	0.1439	0.9987
	60	0.2696	6.9319	1.5108	0.3230	0.9985
Elovich	20	0.2163	47.4033	6.3809	0.5866	0.9920
	30	0.2526	43.7950	7.5420	0.6712	0.9953
	40	0.2654	14.2927	2.8698	0.4012	0.9970
	50	0.2665	9.4590	1.9043	0.2899	0.9980
	60	0.2921	5.1609	1.2527	0.2144	0.9983
Avrami	20	0.3774	148.7241	44.2646	−0.9447	0.9971
	30	0.2273	28.3171	3.3940	0.3819	0.9962
	40	0.3170	6.0240	1.6299	0.2926	0.9957
	50	0.4459	6.6991	2.6695	0.5034	0.9946
	60	0.5538	11.4463	5.0771	1.0813	0.9939

**Table 8:** Equilibrium isotherm parameters of the biosorption process conducted on SPA 5% biosorbent.

Parameter	Freundlich	Temkin	Hill	Redlich- Peterson	Sips	Toth
*K_F_*	4.5154	-	-	-	-	-
*n*	0.7476	-	-	-	-	-
*K_T_*	-	0.8281	-	-	-	-
*b*	-	108.2646	-	-	-	-
*Q_H_*	-	-	96.2335	-	-	-
*K_D_*	-	-	24.0381	-	-	-
*n_H_*	-	-	1.6712	-	-	-
*K_R_*	-	-	-	0.0070	-	-
*a_R_*	-	-	-	−0.9985	-	-
*b_R_*	-	-	-	0.8198	-	-
*Q_S_*	-	-	-	-	96.2335	-
*K_S_*	-	-	-	-	0.1490	-
*B_S_*	-	-	-	-	1.6702	-
*Q_T_*	-	-	-	-	-	6.6691
*a_T_*	-	-	-	-	-	−0.9415
*n_T_*	-	-	-	-	-	−0.000003

**Table 9:** Equilibrium isotherm parameters of the biosorption process conducted on SPRBA 5% biosorbent.

Parameter	Freundlich	Temkin	Hill	Redlich- Peterson	Sips	Toth
*K_F_*	3.2340	-	-	-	-	-
*n*	0.6937	-	-	-	-	-
*K_T_*	-	0.6558	-	-	-	-
*b*	-	98.6681	-	-	-	-
*Q_H_*	-	-	0.0008	-	-	-
*K_D_*	-	-	−0.9998	-	-	-
*n_H_*	-	-	−0.00006	-	-	-
*K_R_*	-	-	-	6.8807	-	-
*a_R_*	-	-	-	3.5347	-	-
*b_R_*	-	-	-	−2.3021	-	-
*Q_S_*	-	-	-	-	64.2878	-
*K_S_*	-	-	-	-	0.1948	-
*B_S_*	-	-	-	-	2.0508	-
*Q_T_*	-	-	-	-	-	5.7159
*a_T_*	-	-	-	-	-	−0.9286
*n_T_*	-	-	-	-	-	0.000006

**Table 10:** Statistical error functions of estimated equilibrium isotherm nonlinear models for the biosorption process conducted on SPA 5% biosorbent.

Equilibrium Isotherm Model	Statistical Error Function				
	*RMSE*	*MPSD*	*HYBRID*	*Χ* ^2^	*R* ^2^
Freundlich	0.5895	2.2028	0.9141	0.027	0.9973
Temkin	0.9787	9.3994	11.0666	0.3417	0.9784
Hill	0.2396	2.2021	0.7805	0.0157	0.9987
Redlich–Peterson	0.3907	3.4869	1.9578	0.0392	0.9965
Sips	0.2396	2.2021	0.7805	0.0157	0.9987
Toth	1.5706	20.8824	48.2691	0.8271	0.9444

**Table 11:** Statistical error functions of estimated equilibrium isotherm nonlinear models for the biosorption process conducted on SPRBA 5% biosorbent.

Equilibrium Isotherm Model	Statistical Error Function				
	*RMSE*	*MPSD*	*HYBRID*	*Χ* ^2^	*R* ^2^
Freundlich	0.5285	5.0164	3.0804	0.0901	0.9936
Temkin	0.7386	6.2446	5.5365	0.1646	0.9876
Hill	1.3477	18.2624	35.5931	0.6222	0.9587
Redlich–Peterson	0.3462	2.9609	1.5882	0.0315	0.9972
Sips	0.3447	3.1836	1.6884	0.0336	0.9972
Toth	1.9571	27.7742	79.9008	1.2840	0.9129

## Data Availability

Not applicable.
